# Combined application of antibiotic bone cement and bone transport technique in tibial osteomyelitis with bone defect: A case report

**DOI:** 10.1097/MD.0000000000050580

**Published:** 2026-09-04

**Authors:** Jie Wang, Bowen Song, Honghao Xu, Shilu Wang

**Affiliations:** a Affiliated Hospital of Shandong University of Traditional Chinese Medicine, Jinan, Shandong Province, China.

**Keywords:** Antibiotic-Loaded Bone Cement, Bone Defect, Bone Transport, Case Report, Tibial Osteomyelitis

## Abstract

**Rationale:**

Management of post-traumatic tibial osteomyelitis accompanied by substantial segmental bone defects (>5 cm) presents a formidable clinical challenge, primarily due to biofilm-associated persistent infections and the inherent complexities of bone regeneration. Single-stage interventions are often insufficient for such complex lesions. This case report demonstrates the value of a staged protocol integrating antibiotic-loaded bone cement (ALBC) with bone transport, which sequentially addresses infection and mechanical bone discontinuity.

**Patient concerns:**

A 54-year-old male presented with chronic tibial osteomyelitis and a 6 cm segmental bone defect following internal fixation for an open fracture.

**Diagnoses:**

Post-traumatic chronic tibial osteomyelitis with a 6 cm segmental tibial bone defect.

**Interventions:**

Staged surgical management was adopted. The first stage included radical debridement, implant removal, infected bone resection, ALBC spacer implantation, and external fixation. After a 3-month interval for infection eradication, the second-stage surgery was performed, consisting of cement removal, antibiotic-impregnated calcium sulfate implantation, and bone transport via distraction osteogenesis at 1 mm/day. Autologous iliac bone grafting was utilized to achieve docking-site union.

**Outcomes:**

Final bony union was achieved at the docking site.

**Lessons:**

This staged approach combining ALBC with bone transport is a viable strategy for complex tibial osteomyelitis with large bone defects. ALBC first creates a sterile and mechanically stable environment, which serves as a critical prerequisite for the success of subsequent reconstructive bone-transport procedures.

## 1. Introduction

Traumatic osteomyelitis is a severe bone and joint infection resulting from exogenous pathogenic microorganisms invading and colonizing bone tissue via traumatic pathways. This infection leads to local inflammatory reactions and bone destruction. Typical clinical manifestations include fever, local swelling, suppuration, and pathological fractures.^[1]^ The tibia is particularly susceptible due to its limited soft tissue coverage and blood supply, resulting in a reported infection rate of 35–40% following open fractures.^[2]^ Once established, the infection often progresses to bone necrosis, pathologically characterized by sequestrum formation and sinus tracts, making surgical intervention the consensus primary treatment for disease control.^[3]^ This article analyzes a successful case managed sequentially with antibiotic-loaded bone cement (ALBC) and bone transport technique. Additionally, it discusses the clinical value of this combined therapeutic approach for complex tibial osteomyelitis with large segmental bone defects (>5 cm), alongside a review of recent literature.

## 2. Case report

**Figure 1.**
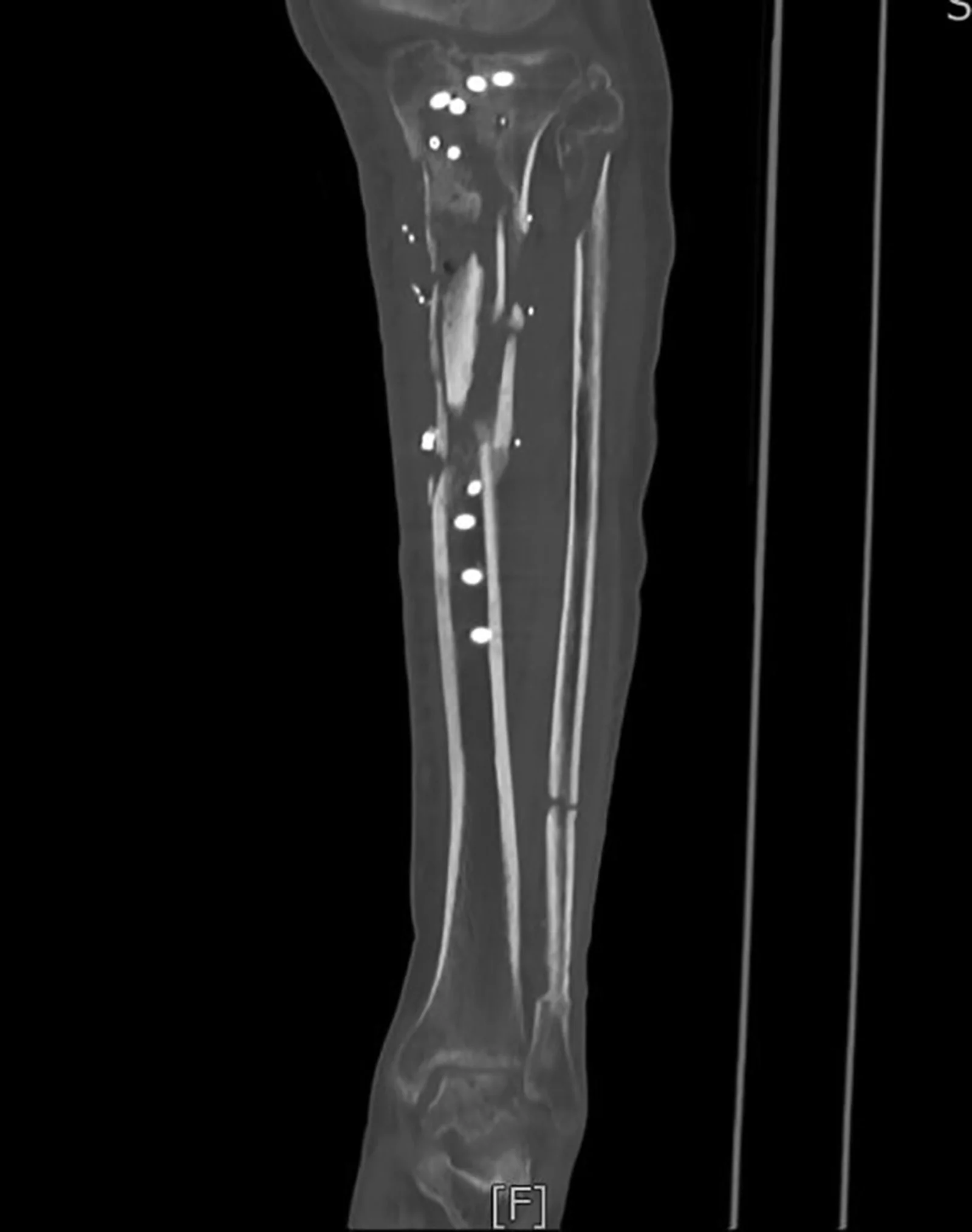
Preoperative imaging upon admission. A computed tomography (CT) scan of the left tibia shows nonunion at the fracture site with surrounding bone atrophy and sclerosis, along with retained internal fixation implants. These findings are consistent with a diagnosis of chronic post-traumatic osteomyelitis.

**Figure 2.**
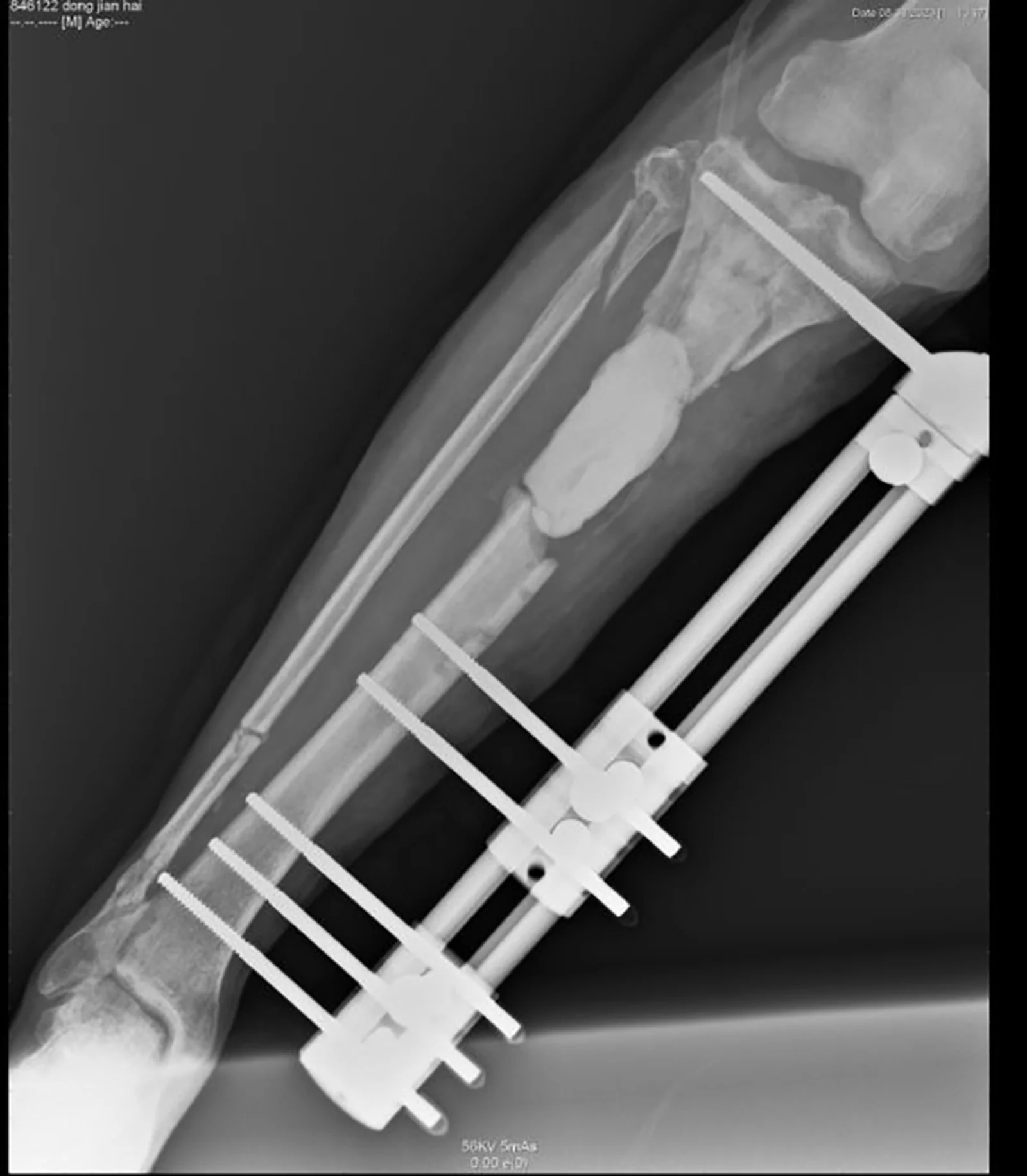
Radiographic appearance after the first-stage surgery. An anteroposterior radiograph demonstrates the application of an external fixator for stabilization. The resected 6 cm segmental bone defect has been filled with an antibiotic-loaded bone cement (ALBC) spacer, which serves to locally elute antibiotics and maintain soft tissue tension.

A 54-year-old male sustained an open left tibiofibular fracture on March 26, 2023, and underwent initial internal fixation, which was followed by postoperative wound breakdown with discharge and bleeding within 20 days. He presented to the Affiliated Hospital of Shandong University of Traditional Chinese Medicine on August 23, 2023. Physical examination revealed multiple surgical scars and a proximal sinus tract with purulent discharge on the left lower leg. Neurological examination showed muscle strength of 0/5 in the left extensor hallucis longus, with normal sensation. Laboratory results showed elevated inflammatory markers (CRP: 43.1 mg/L; ESR: 74 mm/h). CT imaging confirmed nonunion, bone atrophy, sclerosis, and retained implants (Fig. 1). The diagnosis was left tibial traumatic osteomyelitis (Cierny-Mader Type IVB) with a segmental bone defect. After multidisciplinary consultation, a staged surgical treatment plan was developed:

First-stage surgery: On August 31, 2023, the patient underwent removal of internal fixation and radical debridement. Surgical exploration revealed deep osseous involvement, with pale-yellow purulent tissue embedded within sclerotic bone. Subsequent debridement exposed the nonunion ends, demonstrating abundant fibrous scar tissue within the fracture gap and sclerotic, partially necrotic bone. After debridement, the bone ends exhibited satisfactory punctate bleeding, indicating adequate viability of the residual bone for subsequent reconstruction. An external fixator was applied for stabilization using Schanz screws inserted into the distal, proximal, and mid-tibial segments. Following resection of a 6 cm necrotic bone segment and copious irrigation, the defect was filled with an antibiotic-loaded PMMA bone cement block (prepared by manually mixing 40 g of PMMA cement with 4 g of vancomycin) (Fig. 2).

Prior to the second-stage procedure, the decision to initiate bone transport was supported by a comprehensive assessment. Clinically, the patient was afebrile with stable vital signs, and the surgical wounds had healed uneventfully with no local signs of infection. These findings, combined with the marked reduction in inflammatory markers (CRP decreasing from 43.1 mg/L to 13.3 mg/L, ESR from 74 mm/h to 19 mm/h), confirmed adequate infection control and soft tissue readiness for the subsequent procedure.

**Figure 3.**
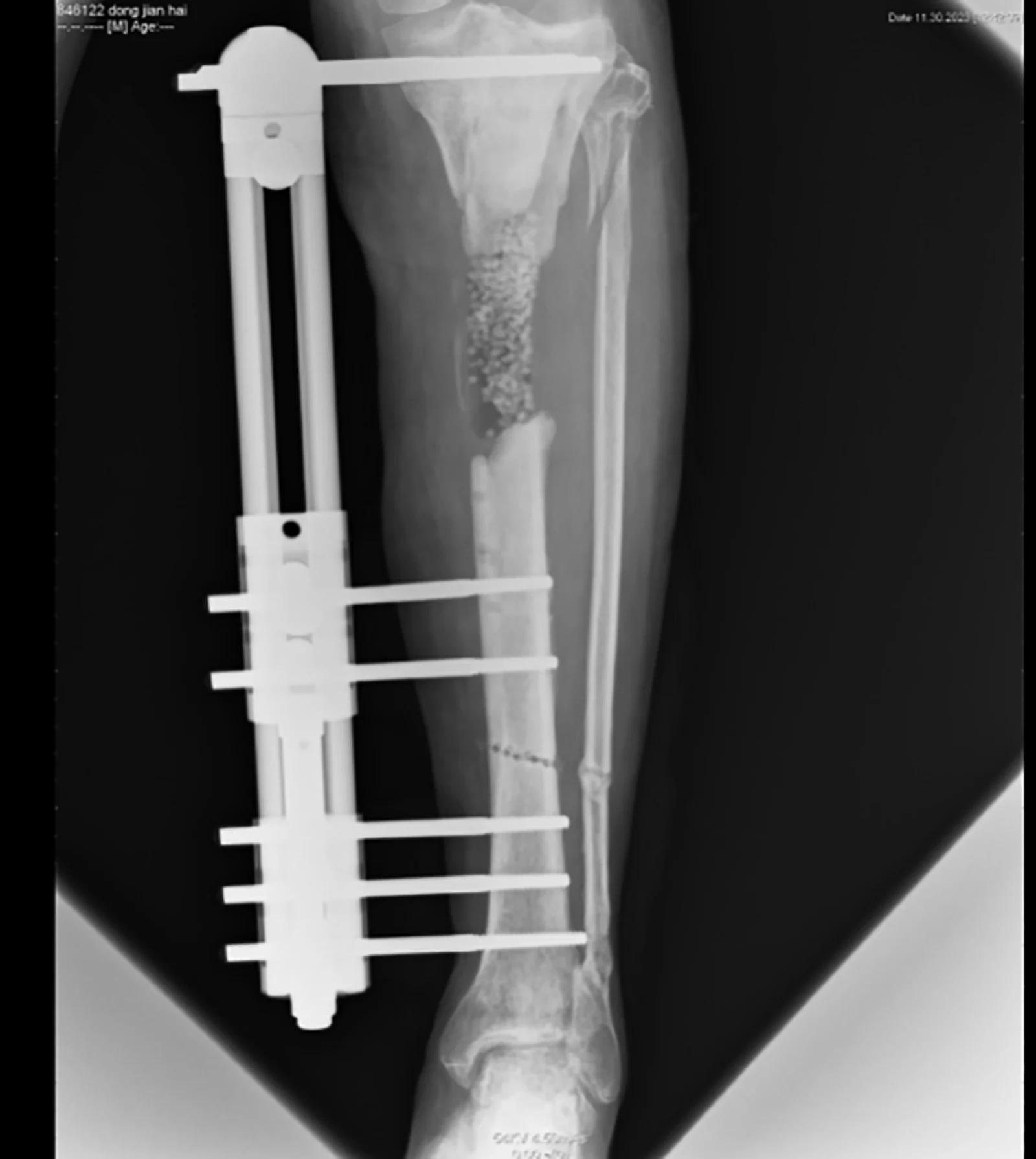
Radiographic appearance after the second-stage surgery. Following the removal of the ALBC spacer, a distal tibial osteotomy has been performed. The bone transport segment has been mobilized, initiating the distraction osteogenesis process to gradually close the bone defect.

Second-stage surgery: On November 30, 2023, the second-stage procedure was performed. The antibiotic bone cement block was removed and replaced with antibiotic-impregnated calcium sulfate granules (prepared at a 10:1 ratio, i.e., 10 g of calcium sulfate per 1 g of vancomycin). A distal tibial osteotomy was then created, and the external fixator was reconfigured to initiate bone transport. The osteotomy site was confirmed to be mobile upon activation of the transport mechanism (Fig. 3). After a 7-day latency period, distraction proceeded at a rate of 1 mm per day.

**Figure 4.**
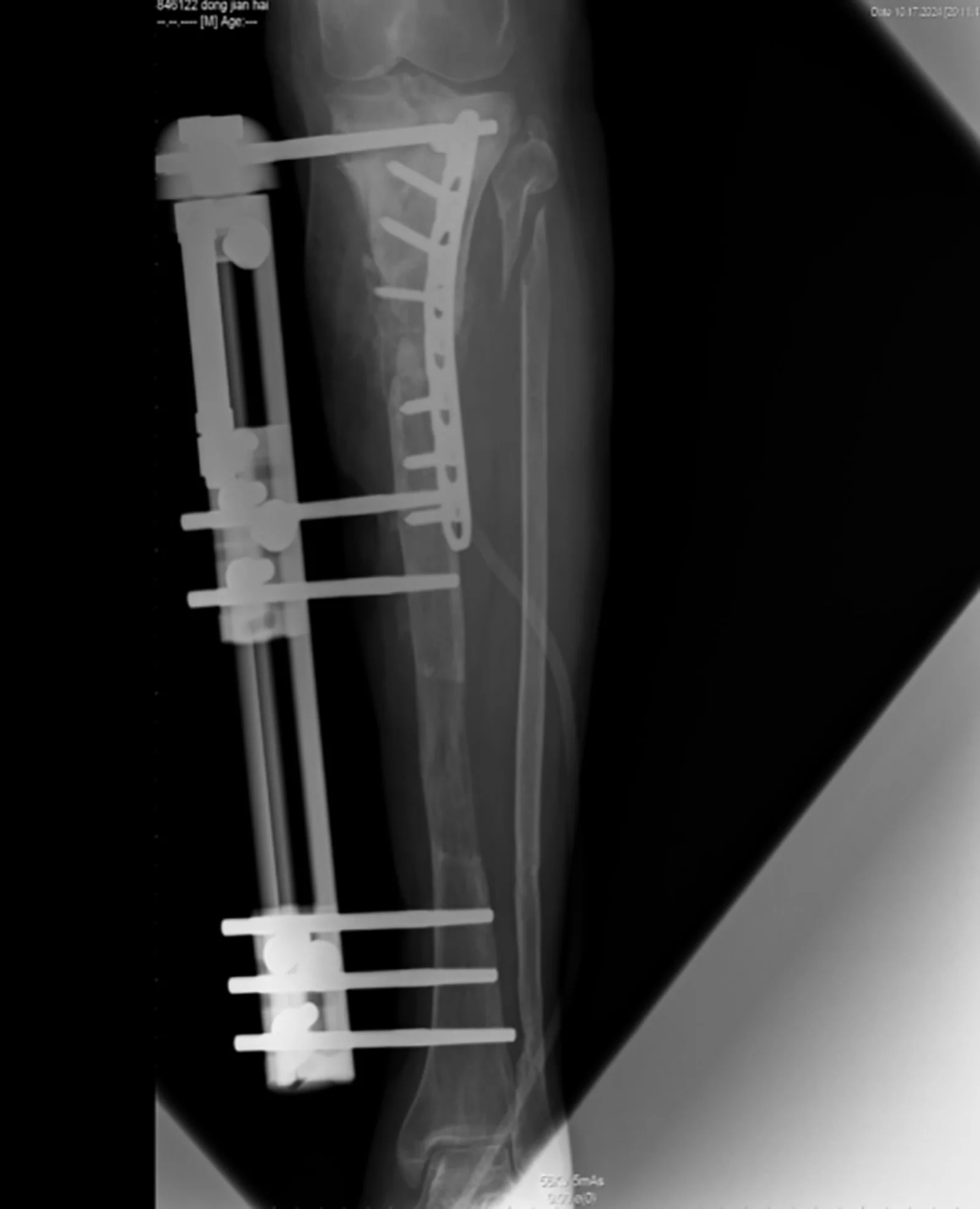
Immediate postoperative radiograph after the final surgery (October 17, 2024). The image shows the status after docking-site revision, autologous iliac bone grafting, and internal fixation. The bone transport segment has successfully reached the distal end, and the regenerate bone in the distraction gap shows satisfactory mineralization.

**Figure 5.**
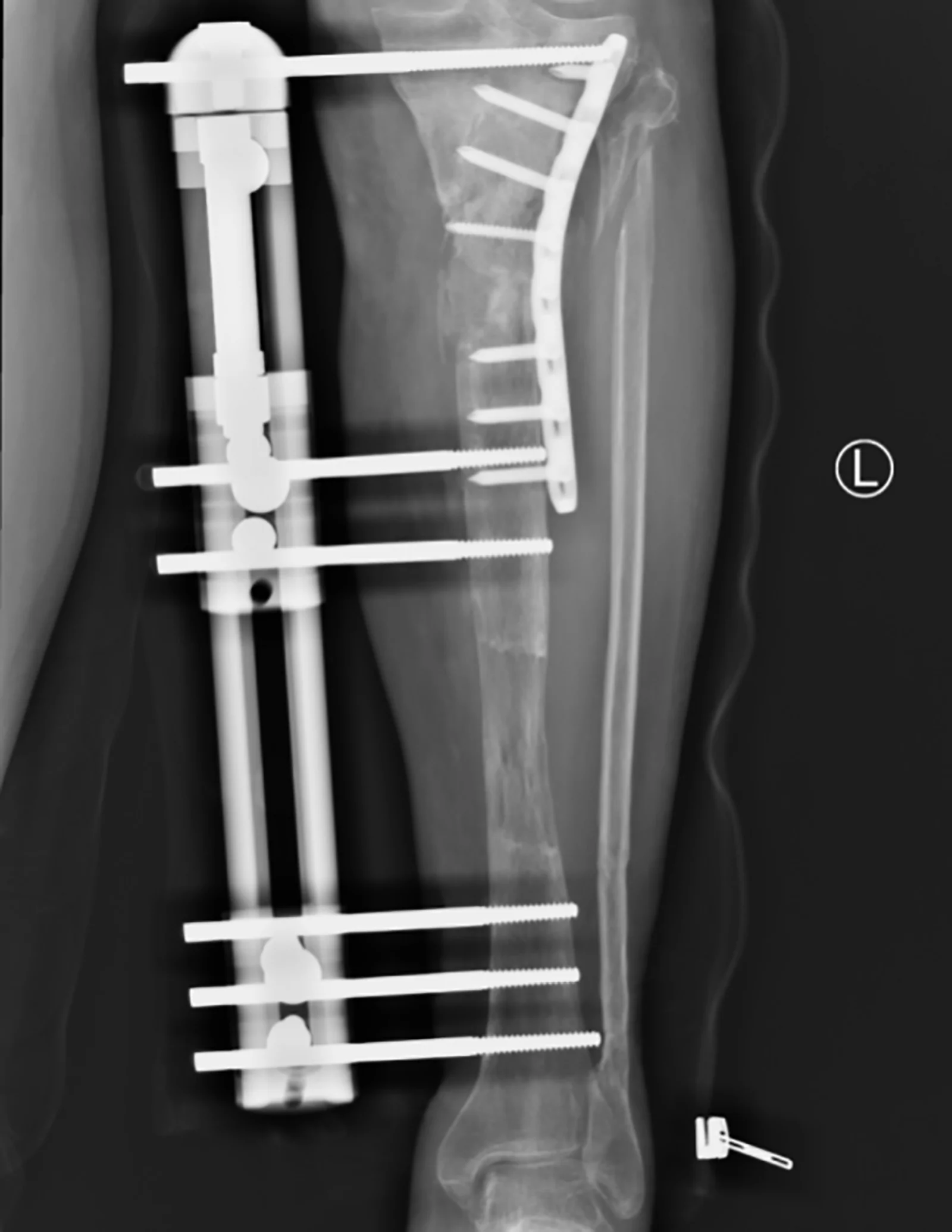
Follow-up radiograph at the latest outpatient visit (December 1, 2024). The fracture line at the docking site is progressively blurring, indicating ongoing consolidation. The regenerate bone in the distraction zone continues to mature with increased density, suggesting favorable bone healing.

Third-stage surgery and follow-up: On October 17, 2024, the patient underwent final docking-site revision with autologous iliac bone grafting and internal fixation (Fig. 4), with subsequent follow-up revealing satisfactory bony consolidation (Fig. 5). At the latest follow-up (February 27, 2025) , the patient was able to ambulate independently without walking aids and reported no resting or weight-bearing pain. No major complications were observed during the entire follow-up period.

## 3. Discussion

Tibial osteomyelitis remains a major challenge in orthopedic trauma, characterized by persistent inflammation, pathological bone remodeling, and biofilm formation.^[4]^ The classification system proposed by Cierny and Mader in 1985,^[5]^ which is based on anatomical extent (Medullary I, Superficial II, Localized III, Diffuse IV) and host physiological status (A: healthy host; B: compromised host; C: treatment worse than disease), provides an important theoretical framework for clinical decision-making. This classification system remains globally recognized in the medical community and is important for clinical diagnosis, accurate assessment of disease severity, and guiding appropriate treatment for osteomyelitis. The management of tibial osteomyelitis faces two core challenges: biofilm-mediated resistance, which impairs antibiotic penetration and host immunity, and segmental bone defects, which require complex regeneration involving revascularization, osteogenesis, and mechanical stability.^[5–7]^ These factors collectively determine the long-term prognosis of osteomyelitis treatment.

The clinical research and application progress of antibiotic bone cement represents a significant advancement in osteomyelitis management. The utility of bone cement as a drug delivery vehicle stems from its ability to provide localized antibiotic release, overcoming both the limited penetration from poor blood supply and the systemic toxicity associated with intravenous administration.^[8,9]^ Beyond drug delivery, ALBC provides crucial temporary mechanical support – without significant property loss at standard doses – to maintain bone alignment, prevent soft tissue contracture, and create optimal conditions for later surgical procedures.^[9–11]^ The one-stage surgical approach involving thorough debridement combined with ALBC implantation is now widely used in osteomyelitis treatment and represents a simple and effective method to manage refractory chronic osteomyelitis.^[12]^

Regarding the clinical research and application of the bone transport technique for bone defects, common surgical treatments include bone grafting, bone transport technique, Masquelet technique, and vascularized free fibular graft transfer. For simple defects smaller than 3 cm, bone grafting can be effective. However, for infected non-unions, chronic osteomyelitis, or defects resulting from segmental resection for malignant tumors, the advantages of bone transport are more pronounced.^[13]^ Previous animal studies^[14]^ demonstrated that the principle of bone transport is distraction osteogenesis. The primary mechanism of this process^[15,16]^ is intramembranous ossification, involving direct expression of Type-I Collagen. It also coordinates complex biochemical signaling pathways through mechanocoupling and biochemical coupling mechanisms, achieving synchronous regeneration of bone and soft tissue to fill the defect. The bone transport technique is now widely used in the treatment of large segmental bone and soft tissue defects due to various causes^[17–20]^ and has achieved favorable clinical outcomes.

The mechanism and advantages of our combined treatment strategy lie in its synergistic effect. As mentioned earlier, the two main features of osteomyelitis are infection and bone defects, with severe infections often resulting in large segmental defects. In infectious diseases like osteomyelitis, pathogens frequently exist as biofilms, which strongly resist host immunity and antimicrobial agents.^[21]^ Performing bone grafting or bone transport under these conditions carries an increased risk of infection. ALBC can effectively disrupt biofilms by releasing high concentrations of antibiotics, providing a prerequisite condition for subsequent procedures. Additionally, bone defects caused by osteomyelitis create an unstable mechanical environment, hindering fracture healing^[22]^ and potentially leading to soft tissue contracture, complicating further treatment. The superior mechanical properties of ALBC offer an effective spacer effect, restore skeletal continuity, provide temporary soft tissue support, and create a stable mechanical environment for subsequent bone transport. Once a favorable environment and temporary support are established, the bone transport technique becomes crucial for successful treatment. Based on the tension-stress principle and intramembranous ossification, it achieves bone regeneration and repair using “local resources,” producing new bone comparable to native bone in structure, mechanical properties, and strength.^[23]^ Therefore, the combination of ALBC and bone transport is a synergistic strategy, where the former creates a sterile and mechanically stable foundation, enabling the latter to successfully achieve biological reconstruction.

## 4. Patient perspective

At the final follow-up, the patient reflected on his treatment journey, affirming the rationale for the staged approach. He stated: “Despite the initial setback and the challenges of the prolonged treatment, especially with the external fixator, the logical sequence of procedures made sense to me. Witnessing the infection clear and the bone gradually regenerate, culminating in the return of my ability to stand and walk, has fully justified the process.” This account not only underscores the patient’s resilience but also powerfully validates the treatment protocol’s effectiveness in achieving its dual goals of infection eradication and excellent functional restoration.

## 5. Conclusion

The staged combined application of antibiotic-loaded bone cement (ALBC) and bone transport may be a viable strategy for selected patients with complex tibial osteomyelitis accompanied by segmental bone defects. This protocol successfully addresses the dual challenges of infection control and structural reconstruction through a synergistic mechanism.

The principal strength of this case lies in the logical, staged execution of this combined mechanical and biological strategy, which aligns with the established surgical principle of first controlling the septic milieu before attempting definitive reconstruction. This approach achieved the dual goals of infection eradication and functional restoration in this complex case. The patient’s perspective further validates the clinical success, highlighting the restoration of walking ability as a key milestone that justified the demanding treatment process.

However, several limitations must be thoughtfully acknowledged. As a single case report, it inherently lacks comparative control and the statistical power to establish generalizable conclusions. The treatment protocol is also highly demanding, requiring specialized equipment, surgical expertise, and exceptional patient compliance over an extended period.

Future research should therefore focus on conducting larger-scale, controlled clinical studies with extended follow-up durations. Such investigations are crucial to validate the long-term efficacy and safety of this combined approach.

## Acknowledgments

The authors thank the patient for agreeing to the publication of this case.

## Author contributions

**Data curation:** Jie Wang.

**Investigation:** Jie Wang.

**Methodology:** Jie Wang.

**Visualization:** Jie Wang, Bowen Song.

**Writing – original draft:** Jie Wang.

**Conceptualization:** Bowen Song, Shilu Wang.

**Supervision:** Honghao Xu, Shilu Wang.

**Writing – review & editing:** Shilu Wang.
